# A reflective learning conversation debriefing model for interprofessional simulation based education

**DOI:** 10.1186/s12909-025-07765-9

**Published:** 2025-10-17

**Authors:** Emad Almomani, John Tobin, Sonia Fernandes, Jacqueline Sullivan, Omar Saadeh, Emad Mustafa, Natalie Pattison, Guillaume Alinier

**Affiliations:** 1 Hamad Medical Corporation/ Hamad International Training Center (HITC), Doha, Qatar; 2 Hamad Medical Corporation (HMC), Doha, Qatar; 3 University of Hertfordshire, School of Health, Medicine and Life Sciences, Hatfield, Hertfordshire, AL10 9AB England, UK; 4 East and North Hertfordshire NHS Trust, Lister Hospital, Coreys Mill Lane, Stevenage, Hertfordshire, SG1 4AB England, UK

**Keywords:** Reflective learning conversation, Debriefing, Simulation based education, Immersive simulation, Advanced life support (ALS), Interprofessional education, Multicultural learning environment

## Abstract

**Background:**

Debriefing for Interprofessional Education (IPE) using Reflective Learning Conversation (RLC) methods enables learners to reflect on their actions, articulate their decisions, and benefit from peer support and the dynamics of group thinking within a team-based context. This study aims to validate a co-designed Reflective Learning Conversation (RLC) debriefing model for use in interprofessional learning groups that vary in professional seniority and clinical experience within a multicultural educational environment. The validation process focuses on enhancing clinical reasoning, clinical judgment, critical thinking skills, and self-efficacy.

**Methods:**

A quasi-experimental pre-test/post-test mixed method. The study sample consisted of a cohort of interprofessional healthcare providers (*n* = 130) who were taking part in European Resuscitation Council (ERC) Advanced Life Support (ALS) courses incorporating Simulation- Based Education (SBE) conducted at Hamad International Training Center (HITC), with the sample equally split between control and experimental groups. Data was collected through subsequent direct observations, validated questionnaires, and focus groups. Descriptive and inferential statistical analyses were performed on the quantitative data, and thematic analysis on the qualitative data.

**Results:**

The experimental group had a significantly higher level of *clinical reasoning*,* judgment*,* and critical thinking skills* compared to the control group at the beginning, midway, and end of simulation activities using the Clinical Reasoning Evaluation in Simulation Tool (CREST) tool, Lasater Clinical Judgment Rubric (LCJR), and Critical Thinking Rubric (CTR). The experimental group scored a significantly higher level of *self-efficacy* than the control group for the Self-Efficacy questionnaire subscales.

**Conclusion:**

Reflective Learning Conversation (RLC) model was found to be valid for enhancing clinical reasoning, clinical judgment, critical thinking, and self-efficacy among interprofessional healthcare providers attending advanced life support simulation-based courses in multicultural learning environments. However, further research is recommended to explore how clinical experience and professional seniority interact with debriefing approaches to influence these cognitive and affective outcomes in simulation-based education.

**Supplementary Information:**

The online version contains supplementary material available at 10.1186/s12909-025-07765-9.

## Introduction

Healthcare providers must demonstrate competence in clinical reasoning, clinical judgment, and critical thinking to reduce errors and enhance patient outcomes [1]. Clinical reasoning is a cognitive process through which healthcare professionals gather and analyse patient information, consider potential diagnoses and treatment options, and make informed decisions [1]. Clinical judgment involves the ability to make sound and timely clinical decisions based on available data and assessment findings [2]. Critical thinking entails active, objective analysis, evaluation, and synthesis of information to reach logical conclusions [3]. It requires challenging assumptions, examining evidence, and considering alternative perspectives before determining a diagnosis or treatment plan [3]. Self-efficacy refers to an individual’s belief in their capacity to perform specific tasks or achieve desired outcomes [4]. Higher levels of self-efficacy are associated with greater persistence, effort, and effectiveness in managing clinical responsibilities and overcoming challenges [4].

As teaching methods have evolved to become more facilitative and learner-centered, the use of post-simulation Reflective Learning Conversation (RLC) debriefing methods has become increasingly common in healthcare Simulation-Based Education (SBE) [5–7]. Following an SBE activity, the goal of facilitating debriefing through an RLC approach is to enable learners to reflect on their actions, articulate their decisions, and draw on peer support and the dynamics of group reasoning within a team context [5, 7]. However, the influence of group harmony during simulation debriefings remains underexplored—particularly within interprofessional learning groups that vary in professional seniority and clinical experience in multicultural environments.

To address this gap, a Reflective Learning Conversation (RLC) debriefing model was co-designed by a working group (*N* = 18) comprising doctors, nurses, researchers, educators, and patient representatives, as described by Almomani et al. (2023) [7]. This co-design process yielded a multiphasic and multimodal RLC debriefing model developed through a theory- and concept-driven approach, supplemented by multiple rounds of expert review. The model integrates Bloom’s Taxonomy [8], appreciative inquiry [9], and the plus/delta method [10] to enhance participants’ clinical reasoning, clinical judgment, critical thinking, and self-efficacy during interprofessional simulation activities. The face and content validity of the model have been established [7]. This paper presents the further validation and testing of the RLC debriefing model for use in Interprofessional Education (IPE) among participants with varying levels of professional seniority and clinical experience in a multicultural learning environment.

## Methods

### Design

A mixed method quasi-experimental pre-test/post-test research design.

### Sample and settings

The participants (*N* = 130), who were equally divided between the experimental and control groups, were enrolled in European Resuscitation Council (ERC) Advanced Life Support (ALS) courses delivered at the Hamad International Training Center (HITC), part of Hamad Medical Corporation (HMC), State of Qatar. The study sample comprised healthcare professionals (*N* = 130) who were required to complete the ALS course as part of their mandatory continuing education. No additional inclusion or exclusion criteria were applied.

A convenience sampling approach was used, selecting participants based solely on their scheduled attendance at ALS courses during the study period. Due to scheduling constraints and operational demands of the ALS course, random allocation was not feasible. Instead, participants were assigned to either the control or experimental group based on their pre-scheduled course dates. Efforts were made to ensure balanced representation across professional roles, clinical departments, genders, and nationalities reflective of the multicultural learning environment.

Each course participant had the opportunity to engage in 18 team- based immersive ALS scenarios, including examination scenarios, which were designed by the ERC as team-based assessments. As the examination scenarios (*n* = 6) were not followed by debriefing, each participant took part in 12 simulation and debriefing sessions. During these sessions, each participant assumed the team leader role exactly three times—once at the beginning, once at the midpoint, and once at the end of the two-day ALS course. Formal assessment was conducted only when participants were acting in the designated team leader role during simulation scenarios.

During the simulation scenarios of the course, participants assigned to the team leader role were expected to follow a structured patient assessment approach using the ABCDE method (Airway, Breathing, Circulation, Disability, Exposure), a standardised framework for assessing critically ill patients in emergency situations [11]. The European Resuscitation Council (ERC) designed the ALS course simulation scenarios to encourage participants to apply a systematic assessment, including the identification of reversible causes of cardiac arrest using the mnemonic “4 Hs and 4 Ts.” These represent: Hypoxia, Hypo-/Hyperkalemia and other metabolic disturbances, Hypothermia, Hypovolemia, Thrombosis, Toxins, Tamponade, and Tension pneumothorax.

The course participants were divided into four groups, each consisting of 4–6 members. Each participant remained in the same learning group throughout the simulation and debriefing activities. Efforts were made to ensure that each group included balanced representation from different backgrounds, professional seniorities, experiences, and nationalities, reflecting real-life interprofessional practice.

Each ALS course faculty included eight valid and certified instructors by the ERC. During the simulation workstations, each group was supported by two facilitators. Due to faculty availability, consistent interprofessional representation of the faculty during each simulation and debriefing session was not always feasible. A ‘follow-the-leader’ co-debriefing approach was adopted, whereby the primary debriefer led the session, and the co-debriefer provided support only when necessary. Each 10-minute ALS scenario was followed by a debriefing session. All groups (experimental and control) followed the same standardised sequence of simulation scenarios to ensure consistency in participant experiences and provide comparable exposure to core ALS content. The only difference between groups was the debriefing model used: the control group received debriefing based on the Norris and Bullock model [5], while the experimental group received debriefing using the Reflective Learning Conversation (RLC) model [7].

### Control group debriefing

Participants in the control group received post-simulation debriefing using the structured model developed by Norris and Bullock [5]. This model supports a guided reflective conversation that begins with a factual recount of events, progresses to exploring the reasoning behind actions and decisions, and concludes with identifying lessons applicable to future practice. Although the model provides a structured framework, it is applied flexibly, allowing facilitators to move between phases based on the evolving discussion. This approach represents the standard debriefing practice in the study setting.

### Experimental group debriefing

Participants in the experimental group engaged in post-simulation debriefing through the Reflective Learning Conversation (RLC) model [7], which was specifically co-designed for this study to address the learning needs of interprofessional groups in multicultural environments with varied clinical experience and professional seniority. The RLC model is a progressive, multimodal, learner-centered approach informed by Bloom’s Taxonomy, Appreciative Inquiry, and Plus/Delta methods. The RLC structure is explicitly co-designed to promote gradual cognitive development, enhance clinical reasoning, judgment, critical thinking, and self-efficacy, and mitigate cognitive overload through incremental reflection and scaffolding over multiple simulations [7].

**Fig. 1:**
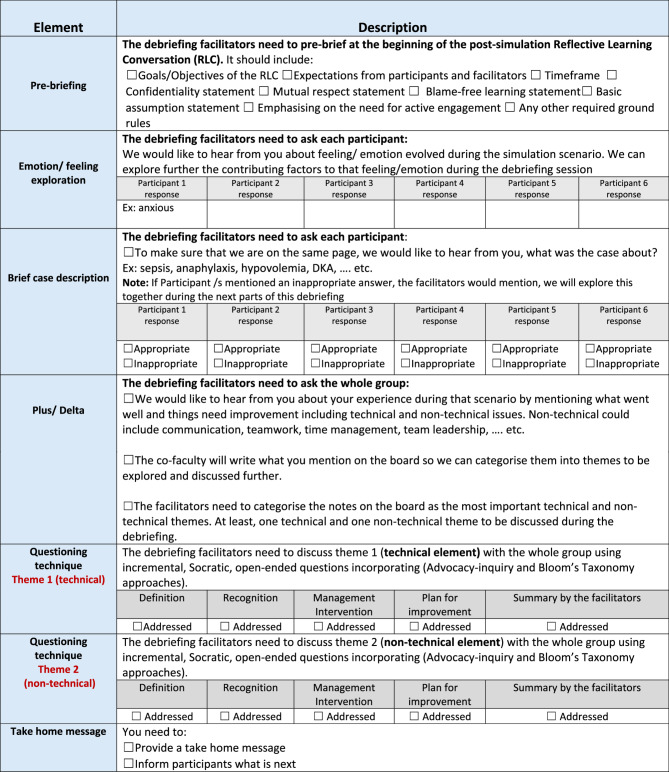
Faculty Reflective Learning Conversation (RLC) debriefing sheet

The ALS instructors who participated in the study as faculty members attended a hands-on practice workshop and completed online training to become familiar with the RLC model debriefing sheet and its delivery format (Fig. 1). They also gained confidence in scoring participants using the observation tools: the Clinical Reasoning Evaluation in Simulation Tool (CREST) [12], the Lasater Clinical Judgment Rubric (LCJR) [13], and the Critical Thinking Rubric [14].

### Data collection

**Fig. 2:**
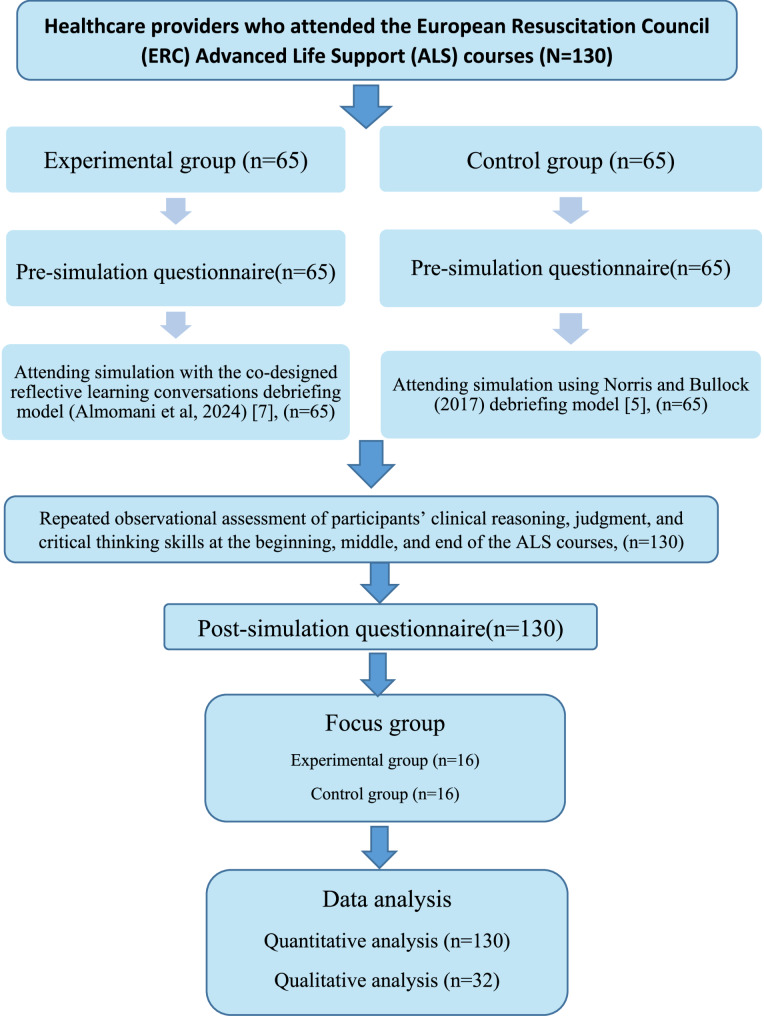
Summary of quantitative and qualitative data collection process and methods

Data was collected through questionnaire, focus group interviews, and direct observations (Fig. 2).

### Direct Observations (Repeated Measures)

Both groups (control and experimental) were evaluated using the same validated assessment tools. The observation tools were the Clinical Reasoning Evaluation in Simulation Tool (CREST) [12], the Lasater Clinical Judgment Rubric Tool (LCJR) [13], and the Critical Thinking Rubric (CTR) [14]. Interrater reliability for the CREST, LCJR, and CTR tools [12–14] was assessed by 12 ALS faculty members, revealing high levels of agreement. The results reflected strong internal consistency and interrater reliability, with Cronbach’s alpha (α) and Intraclass Correlation Coefficients (ICC) of α = 0.968, ICC = 0.972, α = 0.953, ICC = 0.959, and α = 0.853, ICC = 0.859 respectively.

Each participant’s performance as team leader (as described earlier) was assessed in real-time by ALS faculty members using three tools CREST; LCJR; and CTR [12–14]. Assessment criteria for optimal performance in clinical reasoning, judgment, and critical thinking were aligned with the domains measured by these tools and were consistent with ERC standards for ALS performance expectations. The same faculty member rated each participant on all three tools (CREST, LCJR, CTR) during the same observation period. These observations were conducted consecutively during the participant’s assigned team leader role. To ensure consistency and minimise observer bias, the ALS instructors completed the three tools immediately following each observed scenario, before proceeding to the next participant observation.

### General Self-Efficacy (GSE) Questionnaire (Pre-test/Post-test)

Participant’s self-efficacy was evaluated using the self-reported General Self-Efficacy questionnaire [15]. Participants (*N* = 130) completed the GSE questionnaire before attending the course and immediately after completing the course.

### Focus Group

The qualitative arm of this study included four focus groups conducted immediately after the final simulation-based education (SBE) session. Two focus groups included learners randomly selected from the experimental group (*n* = 16), while the other two comprised learners from the control group (*n* = 16). Semi-structured interview guides were used across all sessions to ensure consistency while allowing flexibility for in-depth discussion (Appendix 1 and 2). Focus group data were audio-recorded, transcribed verbatim, and analysed iteratively using Braun and Clarke’s six-step thematic analysis approach to ensure rigour and trustworthiness [16].

A triangulation strategy was employed to integrate the quantitative and qualitative findings [17]. This approach aimed to achieve convergence and complementarity by comparing statistical results with themes emerging from the focus group discussions, thereby enriching the interpretation of outcomes from multiple perspectives. While the primary aim was to explore the impact of the RLC model on the experimental group, data from the control group were also analysed to provide a contrasting perspective on traditional debriefing practices.

### Data analysis

**Fig. 3:**
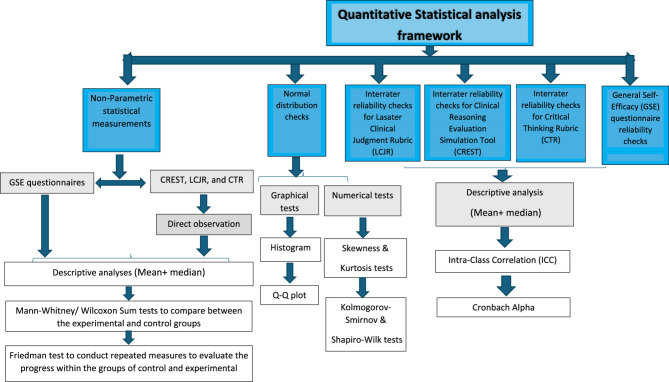
Quantitative Statistical Analysis Framework

Only participants who completed the full course and all required assessments were included in the final analysis. Any participants with incomplete assessments or who did not complete the entire course were excluded prior to data analysis. As a result, there was no missing data in the final dataset. Descriptive and inferential statistical analyses were applied to the quantitative data (Fig. 3). Thematic analysis was performed on the qualitative data.

For the direct observations collected using the CREST, LCJR, and CTR tools, the resulting scores (*n* = 65 per group, per tool, per time point) were analysed for repeated measures and between-group comparisons. The normality of data distribution was assessed for both the experimental and control groups and revealed non-normally distributed data; therefore, non-parametric statistical tests were employed. Within-group comparisons over time were analysed using the Friedman test (a non-parametric test for repeated measures), while between-group comparisons at each time point were analysed using the Mann–Whitney U test. All analyses were conducted using SPSS version 23 (Tables 2 and 4).

For the self-reported General Self-Efficacy (GSE) questionnaire, normality tests also revealed non-normally distributed data in both groups. Accordingly, between-group comparisons were conducted using the Mann–Whitney U test (Table 3).

## Results

Table 1 presents the baseline demographics of experimental and control groups. Both groups included participants from a range of healthcare professions (nursing, medicine, respiratory therapy, and paramedicine), representing diverse backgrounds, levels of seniority, experience, gender, and nationality. Overall, the distribution of participants was reasonably balanced between the experimental and control groups, reflecting the diversity commonly encountered within interprofessional education (IPE) learning environments.

**Table 1:** Demographics of the experimental and control groups

	Experimental Group		Control Group	
**Profession**	Count	Percentage	Count	Percentage
Nurse	18	27.69%	15	23.08%
In-charge nurse	8	12.31%	6	9.23%
Physician - Resident	11	16.92%	19	29.23%
Physician - Specialist	12	18.46%	13	20%
Physician - Consultant	11	16.92%	7	10.77%
Respiratory Therapist	3	4.62%	2	3.08%
Paramedic	2	3.08%	3	4.62%
Total	65	100%	65	100%
**Working Area**	Count	Percentage	Count	Percentage
Accident & Emergency	8	12.31%	13	20%
Medical	11	16.92%	17	26.15%
Critical Care	16	24.62%	10	15.38%
Anesthesia	13	20.0%	9	13.85%
Ambulance Services	2	3.08%	3	4.62%
Respiratory Therapy	3	4.62%	2	3.08%
Surgery	11	16.92%	7	10.77%
Cardiology	1	1.53%	4	6.15%
Total	65	100%	65	100%
**Gender**	Count	Percentage	Count	Percentage
Male	36	55.40	43	66.20%
Female	29	44.60	22	33.80%
Total	65	100%	65	100%
**Years of clinical experience**	Count	Percentage	Count	Percentage
2–5 years	21	32.31%	30	46.15%
6–10 years	19	29.23%	22	33.85%
More than 10 years	25	38.46%	13	20%
**Nationality**	Count	Percentage	Count	Percentage
India	14	21.54	16	24.62%
Philippines	9	13.85	4	6.15%
Jordan	7	10.78	5	7.69%
United Kingdom	5	7.69	4	6.15%
Libya	5	7.69	3	4.62%
Cuba	4	6.15	6	9.23%
Canada	3	4.62	0	0%
Egypt	2	3.07	3	4.62%
Pakistan	4	6.15	5	7.69%
United States	1	1.53	2	3.07%
Ireland	0	0	1	1.53%
Syria	1	1.53	4	6.15%
Qatar	1	1.53	2	3.07%
Tunisia	2	3.07	1	1.53%
Algeria	1	1.53	1	1.53%
Iraq	1	1.53	1	1.53%
Spain	1	1.53	0	0%
Japan	1	1.53	0	0%
Sudan	1	1.53	1	1.53%
Turkey	1	1.53	1	1.53%
France	1	1.53	0	0%
Somalia	0	0	1	1.53%
Iran	0	0	2	3.07%
Yemen	0	0	2	3.07%
Total	65	100%	65	100%

The inferential findings presented in Table 2 indicate that the experimental group consistently scored significantly higher levels of clinical reasoning, judgment, and critical thinking compared to the control group across all three observations. These differences were evident across all three measurement tools: CREST, LCJR, and CTR. The Mann-Whitney U test results showed statistically significant differences between the groups from the second observation onward, with the experimental group showing progressively higher mean ranks than the control group. For the CREST tool, the experimental group achieved higher mean ranks across the first, second, and third observations, with statistically significant differences emerging from the second observation (*p* =.016) and becoming more pronounced at the third observation (*p* <.001). For the LCJR tool, a similar pattern was observed, with significant differences from the second observation (*p* =.002) and increasing significance at the third observation (*p* <.001).

For the Critical Thinking Rubric, statistically significant differences were identified from the second observation (*p* <.001) and further strengthened by the third observation (*p* <.001).

**Table 2:** Descriptive and inferential findings for the direct observations of the control and experimental group participants using CREST*, LCJR*, and (CTR) *

Assessment method	Group (*n* = 65 in each)	Mean Rank	Mann-Whitney U	Wilcoxon W	Z	*p*-Value
1^st^ observation using CREST	Control	60.82	1808.500	3953.500	−1.486	0.137
	Experimental	70.18				
2^nd^ observation using CREST	Control	58.03	1627.000	3772.000	−2.409	0.016
	Experimental	72.97				
3rd observation using CREST	Control	53.28	1318.000	3463.000	−4.165	< 0.001
	Experimental	77.72				
1^st^ observation using LCJR	Control	60.11	1762.000	3907.000	−1.801	0.072
	Experimental	70.89				
2^nd^ observation using LCJR	Control	56.23	1510.000	3655.000	−3.096	0.002
	Experimental	74.77				
3^rd^ observation using LCJR	Control	52.50	1267.500	3412.500	−4.544	< 0.001
	Experimental	78.50				
1^st^ observation using CTR	Control	60.70	1800.500	3945.500	−1.658	0.097
	Experimental	70.30				
2^nd^ observation using CTR	Control	54.98	1428.500	3573.500	−3.446	< 0.001
	Experimental	76.02				
3^rd^ observation using CTR	Control	52.74	1283.000	3428.000	−4.102	< 0.001
	Experimental	78.26				

CREST*: Clinical Reasoning Evaluation in Simulation Tool; LCJR*: Lasater Clinical Judgment Rubric Tool; CTR*: Critical Thinking Rubric

As presented in Table 3, the pre-course comparisons revealed no statistically significant differences between the experimental and control groups across any of the General Self-Efficacy (GSE) subscales (*p* >.05). Post-course comparisons revealed statistically significant improvements in the experimental group across all GSE subscales compared to the control group (*p* ≤.002).

**Table 3:** Pre and post descriptive and Inferential findings of the control and experimental groups for the General Self-Efficacy (GSE) questionnaire

Pre- Course GSE questionnaire								**Post- Course GSE questionnaire**				
**Subscales**	Group	N	Mean Rank	Mann-Whitney U	Wilcoxon W	Z	*P*-Value	Mean Rank	Mann-Whitney U	Wilcoxon W	Z	*P*-Value
1. I can always manage to solve difficult problems if I try hard enough	**Control**	**65**	63.95	2011.500	4156.500	− 0.539	0.590	55.75	1478.500	3623.500	−3.132	0.002
	**Experimental**	**65**	67.05					75.25				
2. If someone opposes me, I can find the means and ways to get what I want	**Control**	**65**	63.58	1988.000	4133.000	− 0.661	0.509	55.75	1478.500	3623.500	−3.132	0.002
	**Experimental**	**65**	67.42					75.25				
3. It is easy for me to stick to my aims and accomplish my goals.	**Control**	**65**	64.15	2024.500	4169.500	− 0.531	0.595	56.36	1518.500	3663.500	−3.311	< 0.001
	**Experimental**	**65**	66.85					74.64				
4. I am confident that I could deal efficiently with unexpected events.	**Control**	**65**	62.46	1915.000	4060.000	−1.042	0.297	55.75	1478.500	3623.500	−3.132	0.002
	**Experimental**	**65**	68.54					75.25				
5. Thanks to my resourcefulness, I know how to handle unforeseen situations.	**Control**	**65**	64.15	2024.500	4169.500	− 0.531	0.595	56.36	1518.500	3663.500	−3.311	< 0.001
	**Experimental**	**65**	66.85					74.64				
6. I can solve most problems if I invest the necessary effort.	**Control**	**65**	62.88	1942.500	4087.500	− 0.894	0.372	55.75	1478.500	3623.500	−3.132	0.002
	**Experimental**	**65**	68.12					75.25				
7. I can remain calm when facing difficulties because I can rely on my coping abilities.	**Control**	**65**	62.01	1885.500	1885.500	−1.193	0.233	56.85	1550.500	3695.500	−2.782	0.005
	**Experimental**	**65**	68.99					74.15				
8. When I am confronted with a problem, I can usually find several solutions.	**Control**	**65**	64.15	2024.500	4169.500	− 0.531	0.595	56.36	1518.500	3663.500	−3.311	< 0.001
	**Experimental**	**65**	66.85					74.64				
9. If I am in trouble, I can usually think of a solution	**Control**	**65**	59.75	1739.000	3884.000	−1.935	0.053	55.75	1478.500	3623.500	−3.132	0.002
	**Experimental**	**65**	71.25					75.25				
10. I can usually handle whatever comes my way.	**Control**	**65**	62.82	1938.500	4083.500	− 0.926	0.355	55.75	1478.500	3623.500	−3.132	0.002
	**Experimental**	**65**	68.18					75.25				

Repeated measures using the Friedman test were performed to examine whether clinical reasoning, judgment, and critical thinking scores changed over time within each group. The results, presented in Table 4, indicated statistically significant changes across the three observations for both the control and experimental groups on all three assessment tools.

**Table 4:** Repeated measures using Friedman test for the direct observations of the control and experimental groups using the CREST, LCJR, and CTR

Control Group				Experimental Group		
	Three direct observations using CREST for the control group	Three direct observations using LCJR for the control group	Three direct observations using CTR for the control group	Three direct observations using CREST for the experimental group	Three direct observations using LCJR for the experimental group	Three direct observations using CTR for the experimental group
N	65	65	65	65	65	65
Chi-Square	75.422	102.069	103.969	88.941	101.743	111.229
Df*	2	2	2	2	2	2
*P*- value	< 0.001	< 0.001	< 0.001	< 0.001	< 0.001	< 0.001

Df*: Degree of freedom; CREST*: Clinical Reasoning Evaluation in Simulation Tool; LCJR*: Lasater Clinical Judgment Rubric Tool; CTR*: Critical Thinking Rubric

### Qualitative FINDINGS

Two themes were derived using thematic analysis: (i) the impact of reflective learning conversation on clinical reasoning, judgment, critical thinking skills, and self-efficacy and (ii) the influencing and contributing factors which enhance clinical reasoning, judgment, critical thinking skills, and self-efficacy while engaging in reflective learning conversations.

The experimental focus group qualitative findings provided important insights that helped explain the observed quantitative improvements in clinical reasoning, clinical judgment, critical thinking, and self-efficacy among participants who engaged in the Reflective Learning Conversation (RLC) model. Participants consistently described how the structured, systematic nature of the RLC debriefing supported their development of key cognitive processes related to clinical reasoning, judgment, and critical thinking. Specifically, Theme 1 highlighted how the RLC model helped participants refine their skills in data collection, intervention prioritisation, and outcome evaluation—critical components aligned with the CREST, LCJR, and CTR assessment tools. Participants attributed their improvements to the opportunity for structured reflection on patient assessment, decision-making, and evaluation processes facilitated by the RLC framework.

*“The reflective learning conversation helped me in developing skills and strategies to collect the most important and relevant patient information*,* so I was able to reason*,* judge*,* and take decisions appropriately.” (Participant 8*,* Focus Group 1)*.

*“The after-simulation reflective learning conversation encouraged me to reflect and consider the most efficient ways and strategies to prioritise patient intervention against the patient assessment findings.” (Participant 4*,* Focus Group 2)*.

Theme 2 provided further explanation of how specific factors within the RLC model contributed to enhanced reasoning, judgment, critical thinking, and self-efficacy. Participants reported that the learner-centered, incremental, and reflective nature of the RLC debriefing fostered deeper understanding and critical analysis of both technical and non-technical aspects of clinical scenarios. They emphasised that these reflective conversations helped them process complex information progressively, mitigating the negative effects of cognitive overload and allowing for meaningful learning without feeling overwhelmed. Furthermore, participants underscored the importance of psychological safety, fostered through structured pre-briefing and emotionally safe debriefing practices, which encouraged open reflection, critical thinking, and risk-taking in advanced thinking.

Additionally, participants identified that working within multicultural, interprofessional groups posed challenges related to communication and engagement but expressed that the structured, inclusive nature of the RLC model helped facilitate equitable participation. This supported the development of clinical reasoning, judgment, and critical thinking with enhanced self-efficacy despite these complexities. Collectively, these qualitative insights reinforce and explain the quantitative findings, demonstrating how the RLC model’s structured, incremental, and learner-centered approach contributed to participants’ cognitive development in these key areas.

*“In our simulation group*,* we had five different nationalities and cultural backgrounds. I think considering the cultural variation of the simulation group by the educator was very important to keep us interacting and engaging in the learning and developing effective clinical reasoning skills and critical thinking skills.” (Participant 1*,* Focus Group 2)*.

*“……*,* and the debriefing reflective discussions helped us to analyse the information and patient findings for deeper understanding. The discussions centered around technical and non-technical skills. That was very helpful to improve my clinical reasoning and judgment skills”. (Participant 5 in focus group 1).*

In contrast to the experimental group, participants from the *control group* described their debriefing experiences as structured but not fully supporting depth, analytical rigour, and reflective value. While debriefing sessions were consistently conducted following each simulation scenario, participants frequently perceived them as superficial in nature. The sessions were described as focused on providing a general overview of the scenario rather than facilitating critical reflection or detailed analysis of clinical actions and decision-making. *“The debriefing felt more like a summary than something that helped me reflect or improve.” (Participant 3*,* Focus Group 3).* Another participant shared, *“There wasn’t really a step-by-step in-depth discussion — we just talked generally to reflect on our performance.” (Participant 2*,* Focus Group 4)*. Furthermore, some control group participants also reported that contributions during debriefings were inconsistent, and that quieter individuals or less experienced staff were less likely to participate meaningfully. *“The hierarchy was present*,* and it made it hard for everyone to feel comfortable contributing equally.” (Participant5*,* Focus Group 3).*

## Discussions

This study aimed to further validate the Reflective Learning Conversation (RLC) debriefing model within Interprofessional Iducation (IPE), particularly in multicultural learning environments comprising learners of diverse professional seniority and clinical experience.

The results indicated statistically significant changes across the three observation points for both the control and experimental groups on all three assessment tools. However, the direction of change differed between the groups. In the experimental group, significant improvements were observed in clinical reasoning, judgment, and critical thinking across all tools. In contrast, the control group demonstrated declines in performance over successive observations. While these findings suggest that the RLC model may provide a structured and effective approach to supporting these learning outcomes, it is important to acknowledge that debriefing effectiveness is influenced by multiple factors beyond the debriefing model alone.

Although the qualitative focus was primarily on exploring the impact of the RLC model, insights from the control group focus discussions provided valuable contrast that helped contextualise the findings. While the control group also participated in structured debriefing sessions, participants described these as general and lacking depth. Their comments highlighted potential limitations of traditional structured debriefing, particularly its lack of explicit focus on promoting critical reflection and advanced cognitive development. Participants reported minimal emphasis on exploring clinical reasoning processes or evaluating outcomes in detail, which may have limited opportunities to develop clinical reasoning, judgment, and critical thinking.

In contrast, focus group participants on the experimental group described the RLC debriefing as systematic, reflective, and learner-centered, enabling them to progressively build skills in clinical reasoning, judgment, and critical thinking. This contrast reinforces the added value of the RLC model in promoting deeper learning through guided, structured conversations. These perspectives suggest that although a debriefing framework was in place, it may not have effectively supported the development of clinical reasoning, judgment, or critical thinking in the same way as the more structured and reflective RLC model used in the experimental group.

The improvements observed in the experimental group may reflect the capacity of the RLC model to assist facilitators in navigating these complexities through a clear framework that guides discussions, encourages participation, and promotes incremental cognitive development [18, 19]. The structured nature of the RLC model appears to empower debriefers to scaffold reflection effectively, guiding participants incrementally through increasingly complex reasoning processes within a psychologically safe environment [20–24]. This pattern is consistent with Vygotsky’s Zone of Proximal Development (ZPD) and the role of structured scaffolding in advancing cognitive capacities [25].

A further possible contributor to the experimental group’s improvement can be the learner-centered and multimodal design of the questioning techniques [26–28]. The integration of Bloom’s Taxonomy, Appreciative Inquiry, and Plus/Delta [8–10], and aimed to create a structured, constructive, and strengths-based reflective environment. Appreciative Inquiry reframes areas for improvement in ways that promote deeper reflection and exploration, encouraging learners to build upon strengths [9]. The progressive questioning structure using Bloom’s Taxonomy—from basic knowledge to higher-order analysis—may have encouraged participation from less experienced learners and supported gradual cognitive engagement for all participants. This aligns with literature highlighting Bloom’s framework as a useful guide for scaffolding learning from lower- to higher-order thinking skills [26, 27, 29]. Moreover, the incremental nature of the RLC model aligns with literature emphasising the value of breaking complex information into smaller, digestible components [28, 29], encouraging self-assessment and reducing cognitive overload [26, 29]. This approach allowed participants to focus on key learning objectives, connect experiences, and construct knowledge gradually [26–29]. Additionally, debriefing effectiveness can be shaped by cultural attitudes toward communication, feedback, and emotional expression [30, 31]. Debriefers must be sensitive to these variations, particularly in multicultural environments, to foster trust and engagement [32]. Structured models like the Reflective Learning Conversation (RLC) appear to mitigate these risks through scaffolding, psychological safety, and inclusive dialogue [30–35].

However, despite potential benefits of multimodal, constructive, and incremental debriefing framework of the RLC model, these potential contributing factors should not be attributed solely to the RLC model but rather to the combined effects of structured debriefing, debriefer competence, learner-related factors such as previous exposure and experience levels before attending simulation activities, in addition to the cultural-related factors such as feedback acceptance and motivation [30, 31, 34, 35]. Acknowledging these intersecting influences strengthens the argument that structured debriefing models like RLC can serve as valuable tools—not standalone solutions—within broader strategies to enhance debriefing effectiveness in complex, multicultural, and interprofessional contexts.

While the potential positive impact of the RLC incremental approach on constructing learning and higher order of thinking, the incremental questioning approach carries risks of rigidity if not adapted to learners’ needs [28, 36]. For example, highly experienced participants may disengage if basic questions dominate discussions, while novice participants might find advanced questions overwhelming [37, 38]. Balancing individual needs with group objectives can be challenging; more experienced participants may disengage if scenarios and debriefing lack complexity, while novices may feel overwhelmed or hesitant to contribute. Power imbalances within groups can limit contributions from less experienced participants [35–38]. Such dynamics can affect engagement and ultimately lead to non-optimal clinical reasoning and critical thinking advancement. Thus, Bloom’s Taxonomy is perhaps best used as a flexible guide rather than a rigid framework by competent debriefers [39]. This highlights the potential role of debriefer competence as a contributing factor to the outcomes observed in both the experimental and control groups. Debriefer competence was not within the scope of this study, pointing to the need for future research to explore the relative impact of facilitator competence versus the debriefing framework on the development of clinical reasoning, judgment, and critical thinking skills.

Furthermore, professional seniority, and clinical experiences variations within the learning groups may also contribute to outcomes observed in both the experimental and control groups. These contributing factors can potentially enrich learning through knowledge sharing and collaborative reasoning [36–38]. Demographics in Table 1 reflected that the experimental group included a higher proportion of participants with more than ten years of clinical experience (38.46%) compared to the control group (20%). Additionally, the experimental group had more participants in senior professional roles, such as in-charge nurses and consultant-level physicians (29.23% vs. 20% in the control group), whereas the control group had a higher proportion of junior staff, including residents (29.23% vs. 16.92% in the experimental group).

This disparity in seniority and experience among participants is noteworthy, as those in more senior professional roles often possess well-developed clinical reasoning schemas, advanced reflective practices, and adaptive expertise [37, 40, 41]. Their leadership responsibilities may also enhance their ability to engage meaningfully in reflective learning and decision-making processes, making them more receptive to the structured format of the Reflective Learning Conversation (RLC) debriefing model [38, 40, 41]. In contrast, the control group’s higher proportion of less experienced and more junior participants may have limited ability to independently sustain reflective practice and develop complex reasoning skills, particularly in the absence of a gradual and constructive debriefing framework [42]. Existing literature suggests that novice and early-career clinicians benefit significantly from guided reflection and feedback, which support the development of their clinical reasoning processes and higher-order thinking skills [42–44].

Therefore, while the greater experience and seniority in the experimental group may have contributed to their improved outcomes compared to the control group, these factors do not diminish the effectiveness of the RLC model. Rather, they underscore the model’s relevance in supporting participants at all levels. Structured debriefing models like RLC may be especially valuable for junior or less experienced staff, who require scaffolded opportunities to build higher reflective capacity and advanced cognitive process. These findings also highlight the need for further research to explore how clinical experience and professional seniority interact with debriefing approaches to influence clinical reasoning, judgment, and critical thinking in simulation-based education.

Moreover, debriefing effectiveness can be shaped by cultural attitudes toward communication, feedback, and emotional expression [30, 32, 33, 42]. Debriefers must be sensitive to these variations, particularly in multicultural environments, to foster trust and engagement [30, 33, 42]. Structured models like the Reflective Learning Conversation (RLC) appear to mitigate these risks through scaffolding, psychological safety, and inclusive dialogue [32, 34, 42]. However, while the RLC model aimed to accommodate cultural diversity, the acceptance of strategies such as open-ended questioning and Appreciative Inquiry may vary across cultures, potentially affecting engagement [30, 33, 34, 42]. These cultural-related aspects were not measured or evaluated in this study, highlighting the need for future research to explore the impact of the RLC model on learner motivation and other culturally related factors.

In summary, the Reflective Learning Conversation (RLC) model appears to enhance clinical reasoning, judgment, and critical thinking in interprofessional simulation-based education. Its gradual, constructive, structured, learner-centred, and psychologically safe approach supports diverse participants in multicultural settings. While findings suggest the RLC model adds value over traditional debriefing methods, outcomes may also be influenced by facilitator competence, cultural related factors, and learner characteristics. Future research should explore how these factors interact to optimise debriefing effectiveness and foster the development of higher-order cognitive skills, including clinical reasoning, judgment, and critical thinking.

### Limitations

- Although the study recognised the potential influence of participant professional seniority and clinical experience, on group dynamics, these variables were not evaluated. Similarly, while the RLC model offered a structured debriefing framework, facilitator competence, style, and adherence to the model were not assessed. As such, the findings cannot fully isolate the impact of the RLC model from group composition or facilitator-related factors.
- The model was tested within the context of the Middle Eastern country with a diverse group of participants. This limits applicability to other contexts at a global level, suggesting the need for a multi-site research study at a global level to enhance the model’s generalisability.
- The RLC debriefing model was tested for use in a face-to-face simulation debriefing setting. This limits applicability for use of the model in Artificial Intelligence (AI) and advanced simulation-based education such as Augmented Reality (AR) and Virtual Reality (VR).
- The study measured immediate post-intervention outcomes without assessing long-term retention or the transfer of learning to clinical practice. The sustainability of improvements in clinical reasoning, judgment, critical thinking, and self-efficacy remains unknown.
- Although validated tools (CREST, LCJR, and CTR) were used, they mainly capture observable behaviours and may not reflect participants’ internal reasoning or metacognitive processes. Future studies should consider methods like cognitive task analysis to better assess these dimensions.

## Conclusion

This study adds to the growing evidence supporting the Reflective Learning Conversation (RLC) model as an effective debriefing approach for enhancing clinical reasoning, judgment, critical thinking, and self-efficacy in interprofessional education (IPE), particularly in multicultural settings with diverse learner backgrounds. The findings suggest that structured, scaffolded debriefing—such as that offered by the RLC model—can help facilitators manage group complexity and foster deeper cognitive engagement through inclusive, progressive reflection.

However, these improvements likely reflect the combined effects of the RLC model, facilitator competence, and learner characteristics, including cultural and experiential diversity. While consistent with literature advocating for structured, multimodal debriefing, the results emphasise the importance of adapting debriefing strategies to context-specific learner needs and dynamics.

Further research is needed to better understand the interplay between facilitator skill, learner diversity, cultural influences, and the sustained impact of structured debriefing on clinical reasoning, judgment, and critical thinking.

## Supplementary Information

Supplementary Material 1.

Supplementary Material 2.

## Data Availability

The datasets used and/or analyzed during the current study are available from the corresponding author on reasonable request.
