# Supplementary material for: A reflective learning conversation debriefing model for interprofessional simulation based education
Source: BMC Med Educ. 2025 Oct 17;25:1434. doi: 10.1186/s12909-025-07765-9 (PMC12533328; doi:10.1186/s12909-025-07765-9)
Supplement: Supplementary file 1 — Supplementary Material 1. [file 12909_2025_7765_MOESM1_ESM.pdf]

Focus group interview questions- intervention group

- Do you think the Post- simulation Reflective Learning Conversation(RLC) is an effective way of learning and teaching while attending simulation-based courses? why?

- Do you think Post- simulation Reflective Learning Conversation(RLC) is an effective method to enhance clinical reasoning, judgment, critical thinking, and self-efficacy while attending simulation-based courses? why?

- Do you think using a structured model of Post- simulation Reflective Learning Conversation(RLC) while attending simulation-based courses could enhance your enhance clinical reasoning, judgment, critical thinking, and self-efficacy? why?

- Do you think the Post- simulation Reflective Learning Conversation(RLC)model which was implemented in your simulation-based courses enhanced your clinical reasoning, judgment, critical thinking, and self-efficacy to optimal levels? Why?

- Do you think the Post- simulation Reflective Learning Conversation(RLC) method you attended optimized your clinical reasoning, judgment, critical thinking, and self-efficacy considering a group variation based on; different specialties, seniority, case complexity, and competence levels? why?

- Do you think the Post- simulation Reflective Learning Conversation(RLC) method you attended helped you to avoid getting cognitive overload? why?

Institutional Review Board Hamad Medical Corporation Doha - Qatar Hamad

MRC-01-23-606 Validity: 12 12 2024 - 11 12 2025 E-stamped 12 Dec 2024

FOCUS GROUP INTERVIEW QUESTIONS- INTERVENTION GROUP

- What do think about the impact of the questioning technique the facilitator used on your clinical reasoning, judgment, critical thinking, and self-efficacy enhancement? Why

- Do you think there are limitations and challenges to using the Post-simulation Reflective Learning Conversation(RLC)model to optimize your clinical reasoning, judgment, critical thinking, and self-efficacy skills? If yes, can you explain?

- How do think a Post- simulation Reflective Learning Conversation(RLC)model can be enhanced and improved to optimize clinical reasoning, judgment, critical thinking, and self-efficacy considering; specialty, seniority, simulation case complexity, and competence levels while attending simulation-based courses?

Clinical Reasoning: Clinical reasoning is the cognitive process healthcare professionals use to gather and analyze patient information, assess potential diagnoses and treatment options, and make informed clinical decisions. It involves synthesizing knowledge from various sources, such as patient history, physical examinations, laboratory tests, and medical literature, to develop a comprehensive understanding of a patient's condition and determine the most appropriate course of action.

Clinical Judgment: Clinical judgment refers to the ability of healthcare professionals to make sound and timely decisions in clinical settings based on their clinical reasoning skills. It involves drawing conclusions, making predictions, and evaluating the effectiveness of interventions while considering the unique circumstances and needs of individual patients. Clinical judgment is essential for providing safe and effective patient care.

Critical Thinking: Critical thinking is the process of actively and objectively analyzing, evaluating, and synthesizing information to make reasoned and logical decisions. It

Institutional Review Board Hamad Medical Corporation Doha - Qatar حمد Hamad

MRC-01-23-606 Validity: 12 12 2024 - 11 12 2025 E-stamped 12 Dec 2024

involves questioning assumptions, examining evidence, and considering alternative perspectives before arriving at a conclusion. In healthcare, critical thinking is crucial for solving complex problems, assessing patient needs, and making informed decisions about diagnosis and treatment.

Self-Efficacy: Self-efficacy refers to an individual's belief in their own ability to accomplish specific tasks or achieve particular goals. It is a self-assessment of one's competence and confidence in performing particular actions. In healthcare, self-efficacy can influence a healthcare professional's motivation, behavior, and performance. Higher levels of self-efficacy are often associated with greater persistence, effort, and success in managing clinical responsibilities and challenges.

Institutional Review Board Hamad Medical Corporation Doha - Qatar

MRC-01-23-606 Validity: 12 12 2024 - 11 12 2025 E-stamped 12 Dec 2024
