# Supplementary material for: A reflective learning conversation debriefing model for interprofessional simulation based education
Source: BMC Med Educ. 2025 Oct 17;25:1434. doi: 10.1186/s12909-025-07765-9 (PMC12533328; doi:10.1186/s12909-025-07765-9)
Supplement: Supplementary file 2 — Supplementary Material 2. [file 12909_2025_7765_MOESM2_ESM.pdf]

FOCUS GROUP INTERVIEW QUESTIONS- CONTROL GROUP

- Do you think reflective learning conversation is an effective way of learning and teaching while attending critical care simulation-based courses? why?

- Do you think the customary simulation-based reflective learning conversation method (debriefing /feedback) is an effective method to enhance clinical reasoning, judgment, critical thinking, and self-efficacy while attending critical care simulation-based courses? why?

- Do you think the customary simulation-based reflective learning conversation method (debriefing /feedback) which was implemented during the simulation activities was effective to optimize your clinical reasoning, judgment, critical thinking, and self-efficacy? why?

- How do think the customary simulation-based reflective learning conversation method (debriefing /feedback) which was implemented during your simulation activities can be enhanced and improved to optimize your clinical reasoning, judgment, critical thinking, and self-efficacy while attending critical care simulation-based courses?

- Do you think the customary simulation-based reflective learning conversation method (debriefing /feedback) you attended optimized your clinical reasoning, judgment, critical thinking, and self-efficacy considering a group variation based on; different specialties, seniority, case complexity, and competence levels? why?

- How do you think the next simulation courses can be improved to enhance your clinical reasoning, judgment, critical thinking, and self-efficacy skills

Institutional Review Board
Hamad Medical Corporation - Doha - Qatar
حمد
Hamad

MRC-01-23-606 Validity: 12 12 2024 - 11 12 2025 E-stamped 12 Dec 2024

considering specialty, seniority, case complexity, and competence factors?

Clinical Reasoning: Clinical reasoning is the cognitive process healthcare professionals use to gather and analyze patient information, assess potential diagnoses and treatment options, and make informed clinical decisions. It involves synthesizing knowledge from various sources, such as patient history, physical examinations, laboratory tests, and medical literature, to develop a comprehensive understanding of a patient's condition and determine the most appropriate course of action.

Clinical Judgment: Clinical judgment refers to the ability of healthcare professionals to make sound and timely decisions in clinical settings based on their clinical reasoning skills. It involves drawing conclusions, making predictions, and evaluating the effectiveness of interventions while considering the unique circumstances and needs of individual patients. Clinical judgment is essential for providing safe and effective patient care.

Critical Thinking: Critical thinking is the process of actively and objectively analyzing, evaluating, and synthesizing information to make reasoned and logical decisions. It involves questioning assumptions, examining evidence, and considering alternative perspectives before arriving at a conclusion. In healthcare, critical thinking is crucial for solving complex problems, assessing patient needs, and making informed decisions about diagnosis and treatment.

Self-Efficacy: Self-efficacy refers to an individual's belief in their own ability to accomplish specific tasks or achieve particular goals. It is a self-assessment of one's competence and confidence in performing particular actions. In healthcare, self-efficacy can influence a healthcare professional's motivation, behavior, and performance. Higher levels of self-efficacy are often associated with greater persistence, effort, and success in managing clinical responsibilities and challenge

Institutional Review Board
Hamad Medical Corporation Doha - Qatar
حمد Hamad

MRC-01-23-606 Validity: 12 12 2024 - 11 12 2025 E-stamped 12 Dec 2024
